# Exercise Alters *FBF1*-Regulated Novel-miRNA-1135 Associated with Hydrolethalus Syndrome 1 in Rheumatoid Arthritis: A Preliminary Study

**DOI:** 10.2174/0122115366294831240606115216

**Published:** 2024-07-03

**Authors:** Vimolmas Tansathitaya, Witchana Sarasin, Tanapati Phakham, Vorthon Sawaswong, Prangwalai Chanchaem, Sunchai Payungporn

**Affiliations:** 1 College of Sports Science and Technology, Mahidol University, Phutthamonthon Sai 4 Rd, Salaya, Phutthamonthon District, Thailand;; 2 Center of Excellence in Systems Biology, Faculty of Medicine, Chulalongkorn University, 1873 Rama 4 road, Pathumwan, Bangkok, 10330, Thailand;; 3 Center of Excellence in Systems Biology, Faculty of Medicine, Chulalongkorn University, 1873 Rama 4 road, Pathumwan, Bangkok, 10330, Thailand;; 4 Research Unit for Systems Microbiology, Department of Biochemistry, Faculty of Medicine, Chulalongkorn University, 1873 Rama 4 Rd, Pathum Wan, Bangkok, 10330, Thailand;; 5 Research Unit for Systems Microbiology, Department of Biochemistry, Faculty of Medicine, Chulalongkorn University, 1873 Rama 4 Rd, Pathum Wan, Bangkok, 10330, Thailand;; 6 Research Unit for Systems Microbiology, Department of Biochemistry, Faculty of Medicine, Chulalongkorn University, 1873 Rama 4 Rd, Pathum Wan, Bangkok, 10330, Thailand

**Keywords:** Novel, miRNA, exercise, hydrolethalus syndrome, rheumatoid arthritis, autosomal

## Abstract

**Background:**

Hydrolethalus Syndrome 1 (HYDS1) is a rare disorder that occurs commonly in Finnish infants but originates from the mother. This autosomal recessive syndrome is associated with the *FBF1,* which is usually expressed in the centriole. The *FBF1* is an inheritable arthritis disease phenotype that includes rheumatoid arthritis. Several studies have investigated males with *FBF1* mutation carriers also related to arthritis diseases, including those under rheumatoid arthritis conditions, which revealed the possibility of conferring the gene mutation to the next generation of offspring. Nonetheless, there are some complications of *FBF1* mutation with target miRNAs that can be affected by exercise.

**Objective:**

The objective of this study was to evaluate the different exercises that can be utilized to suppress the *FBF1* mutation targeted by Novel-rno-miRNAs-1135 as a biomarker and assess the effectiveness of exercise in mitigating the *FBF1* mutation.

**Methods:**

Four exercise interventional groups were divided into exercise and non-exercise groups. One hundred microliter pristane-induced arthritis (PIA) was injected at the dorsal region of the tails of rodents and introduced to the two PIA interventional groups. On day forty-five, all animals were euthanized, and total RNA was extracted from the blood samples of rodents, while polymerase chain reaction (PCR) was amplified by using 5-7 primers. Computerization was used for miRNA regulation and analysis of target gene candidates.

**Results:**

The novel-rno-miRNA-1135 was downregulated to *FBF1* in exercise groups. The exercise was found to have no significant impact in terms of change in novel-rno-miRNA-1135 regulation of *FBF1* expression.

**Conclusion:**

Exercise has no impact on novel-rno-miRNA-1135 targeted for *FBF1* in autosomal recessive disease.

## 1 INTRODUCTION

Congenital Hydrolethalus Syndrome 1 (HYDS1) may impact fatality and is common among Finnish newborns. At present, HYDS1 syndrome exhibits a low prevalence and limited global recognition. The National Public Health Institute and National Institute of Health and Welfare in Finland have reported an incidence rate of approximately 1 in 20,000 newborns with HYDS1 [1]. Furthermore, isolated cases of HYDS1 identification in embryos and infants have been reported in a small number of countries, including Brazil, France, and Thailand [2-4]. Some newborns survived for up to 6 days after commencement of labor [1], while some pregnancies had gestation periods varying from 12 to 40 weeks, and others involved induced abortions [1]. This autosomal recessive syndrome is expressed in the 11q24.2 chromosome with clinical characteristics involving polydactyly, central nervous system defects, and brain ventricles vulnerable to subarachnoid space, making the hydrocephalus external. In this syndrome, the foramen magnum is keyhole-sculpted, with polydactyly on both the hands and feet, clubbed feet, small and poorly formed mandible nose, hypoplastic eyes, large atrioventricular heart defects, airway stenosis, and abnormal lung lobation [5]. The female fallopian tube contains cilia, mostly in the ampulla uterine tube, which pass on signaling to mediate cell-cell communication and can cause a variety of ciliopathies in progeny [6]. The syndrome is expressed *via* gene expression that passes to male and female offspring from carrier parents in terms of polymorphism in linkage disequilibrium [7]. Currently, linkage disequilibrium has been undertaken in animal studies for specific species but has not yet been broadly used for animal research [8]. This study focused on identifying the risk factors for the syndrome in rodents, using biomarkers in gene expression and miRNAs as markers to predict the syndrome in subsequent generations of offspring [9].

The most frequent factors were handed down to the offspring and identified for HYDS1 [7]. The *FBF1* (Fas Binding Factor 1) gene is a PUF protein (Pumilio and FBF). The protein participates in the meiotic cell cycle and promotes cell growth. The *FBF1* is a remarkably similar regulator of the PUF protein family in *C. elegans* [10], primarily linked to HYDS1 in mammals, and is found in the miotic centriole, sperm centriole, centrosome, spindle pole, and part of the ciliary transition fiber (TFs) [11]. This protein is also linked to a variety of mammalian phenotypes, including increased immune system activity, unusual stationary movements, and atypical CD4 and NK-T cell counts (International Mouse Phenotyping Consortium). Gene mutations can be passed down through the germline and somatic cell expansion [12].

In mammals, *FBF1* induces ciliopathies and syndromic disorders, leading to diseases, such as neuronal maturation, tumorigenesis, and HYDS1 [13]. *FBF1* induces protein docks to various protein compartments, such as transition fibers (TFs) and transition zones (TZs). These genes comprise *FBF1* and are regulated by the ciliary gate [14]. On the other hand, many studies have suggested that *FBF1* docks to TF functions and initiates ciliogenesis [15]. *FBF1* also binds to the TF dysfunction zone, leading to pathological human phenotypic malformation. However, this mechanism requires further experimentation [15].

The gene in *C. elegans* also usually controls the sperm/oocyte, but gene mutation is generally self-fertile; this protein regulates mRNAs that inhibit translation and degrade mRNA transcription. Furthermore, *FBF1* cannot be depleted by interference RNA (iRNA) in *C. elegans* [16]. By contrast, a study in mammals found that silencing of the *FBF1* gene could be regulated by 20-22 nucleotides and post-transcriptional small non-coding microRNAs, which might affect cell proliferation in prophase I [17]. The miRNA markers and target gene candidates can be used as biomarkers for disease prognosis in mammals and they can be used to predict disease phenotypes [18].

Regulation of the *FBF1* and miRNAs are biomarkers in many diseases. One study mentioned that the inheritance of the rare HYDS1 disease has also been investigated using miRNAs and gene regulation as markers (Gene Cards: The Human Genes Database). Many studies have also suggested that ciliopathic disease is carried by mothers, while others suggested that the disease originated from *FBF1* gene mutation. Furthermore, some authors mentioned that the *FBF1* gene is related to HYDS1 inheritance and is also applicable to the diagnosis of other phenotypic immunity system diseases, such as leukocytes and increased cytokine cells [19-21].

Many studies on epigenetics have mentioned the benefits of exercise on changes in human molecular mechanisms, with similarities between the *FBF1* gene and miRNA regulation showing that exercise and an active lifestyle can influence the process of epigenetic and genomic modifications [22, 23]. Exercise involves various locomotion movements that improve energy expenditure. Exercise also increases protein expression and changes protein activation in molecular and cellular processes [22]. Signaling during exercise activates multiple pathways and modifies transcription, translation, post-transcription, and post-translation [22].

Post-transcriptional miRNAs regulate target genes and are also regulated by gene expression depending on lifestyle and the environment. In general, the mi-RNAs and target genes can interact to benefit exercise in many systems, including the immune system [24]. Moreover, the study on exercise and physical mechanism change has been widely known for decades. However, a niche study area related to exercise and inheritable genetic disease in autosomal recessive syndromes has not received adequate attention. Consequently, several underlying hypotheses suggest that an autosomal recessive inheritance syndrome, such as HYDS1 in a male carrier of *FBF1* mutation, may transfer the mutation to progeny with the presence of a rheumatoid arthritis carrier. Thus, the goals of this research were to **1**) assess whether active or sedentary lifestyles had any impact on the inhibition of recessive *FBF1* risk to HYDS1 transmitted to next-generation progeny, **2**) examine the inherited risk of *FBF1* to HYDS1 being transmitted to progeny, whether from a normal father who exercised or a normal father who did not exercise, and **3**) examine the risk of *FBF1* to HYDS1 being inherited to offspring, whether from a father who exercised and had rheumatoid arthritis or a father without exercise who had rheumatoid arthritis. The findings of this study could potentially be considered as part of future family health promotion guidelines for family planning consultations in primary care [25]. This would raise awareness of HYDS1 risks and empower couples to make informed decisions about their reproductive health.

## 2 MATERIALS AND METHODS

### 2.1 Animals

**Fig. (1):**
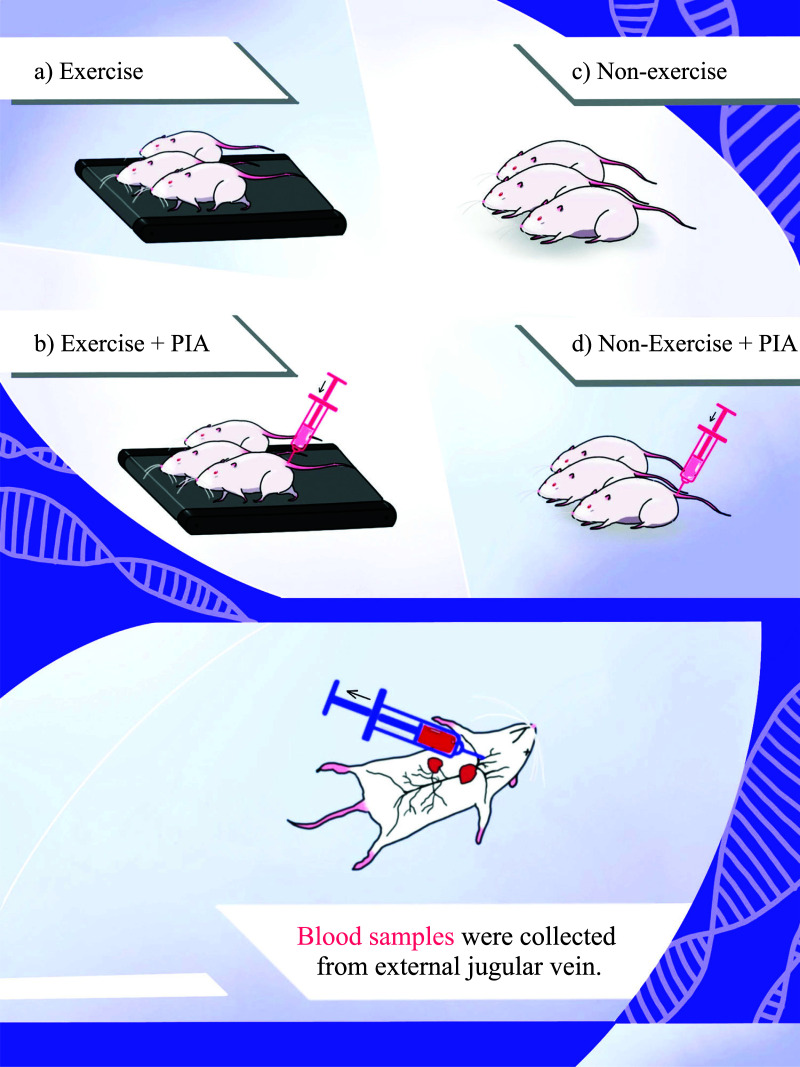
**(a-d)** Exercise interventions and blood sample collections.

The study protocol, including euthanasia, followed the animal care guidelines given by the Association for Assessment and Accreditation of Laboratory Animal Care International (AAALAC International), and the Chulalongkorn University Animal Care and Use Committee approved the study. Twelve healthy male Sprague-Dawley rats aged 11 weeks old were purchased from Nomura Siam International (Bangkok, Thailand) and placed in four cages, with three simple randomized rats housed in each cage. The sample size for a pilot study was determined based on the findings of Sophie *et al*. (2023) [26]. The rats then received an injection into the dorsal region of the tail containing 100 µl of pristane-induced arthritis (PIA) [27, 28]. They were assigned to four exercise groups, including **1**) an exercise group without PIA, **2**) a non-exercise group without PIA, **3**) an exercise group with PIA, and 4) a non-exercise group with PIA. Living conditions were controlled to provide a 24-hour cycle (12 h of darkness and 12 h of light) at 22°C with food and water supplied ad libitum (Fig. **1**). The animals were obtained from Chulalongkorn University Laboratory Animal Center.

### 2.2 Exercise Intervention

**Fig. (2):**
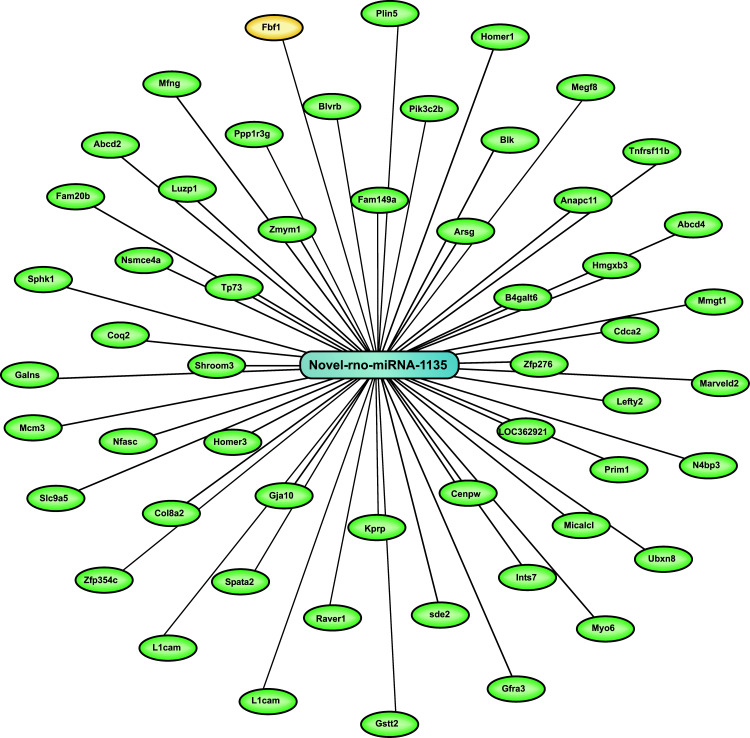
Novel-rno-miRNAs- 1135 and target gene candidates.

The rats were quarantined for two weeks and then subjected to treadmill training for a further week before being separated into four groups, each containing three specimens. Group 1 comprised non-exercise without PIA induction (N-EX), while Group 2 was non-exercise with PIA induction (N-EX+PIA), Group 3 was exercise without PIA induction (EX), and Group 4 was exercise with PIA induction (EX+PIA), as shown in Fig. **2**. The exercise consisted of using a high-speed treadmill for one hour at 20-25 m/min. The weights of the rats were measured at 2-day intervals. The rats that exercised were permitted 15 min of rest on occasions when they exhibited tiredness and were unwilling to run in the absence of stimulation *via* sound or electricity. After 45 days, the rats were euthanized using CO_2_.

### 2.3 Sample Collection

EDTA blood samples were obtained from the external jugular vein, as shown in Fig. **2**. A centrifuge was used for 8 min to separate the plasma. Aliquots were collected and stored at -80°C until required. Total RNA was extracted using TRIzol Reagent and miRNeasy Mini Kit (Qiagen) before quantification and qualification using an Agilent 2100/2200 Bioanalyzer (Agilent Technologies, Palo Alto, CA, USA), NanoDrop (Thermo Fisher Scientific Inc.), and 1% agarose gel electrophoresis. Finally, a GenTegra tube under vacuum was used to dry the RNA samples.

### 2.4 Preparation of the Small RNA Library

Ligation of the 3´SR adaptor from Illumina to the small RNA was performed using the 3´ligation enzyme. The excess 3´-SR adaptor was hybridized with the SR RT primer to ensure that adaptor dimer formation did not occur. Ligation of the 5´SR adaptor from Illumina to the small RNA was carried out using the 5´ligation enzyme, while the synthesis of first-strand cDNA was performed using ProtoScript II reverse transcriptase. P5 and P7 primers were then used to amplify the samples *via* PCR. The primers contained sequences that served to anneal with the flow cells to bridge PCR, and the P7 primer carried a six-base index, which permitted multiplexing. The resulting PCR products of ~150 bp were collected and purified by polyacrylamide gel electrophoresis (PAGE). An Agilent 2100/2200 Bioanalyzer (Agilent Technologies, Palo Alto, CA, USA) was used to validate the final DNA library before quantification using a Qubit 3.0 Fluorimeter (Invitrogen, Carlsbad, CA, USA).

### 2.5 Sequencing of Small RNA

Various libraries with different indices were initially multiplexed and subsequently loaded into HiSeq (Illumina, San Diego, CA, USA). Sequencing was performed using a 2×150 paired-end (PE) configuration, while image analysis and base calling were performed using HiSeq Control Software (HCS) + OLB + GAPipeline-1.6 (Illumina) with HiSeq Instrument image analysis.

### 2.6 Data Analysis

FastQC software (v0.10.1) was used to verify the quality of the FASTQ sequences. The raw sequencing reads underwent adaptor trimming and quality filtering (<Q30) using Trimmomatic (v0.30) [29]. After cleaning, the reads were matched to the reference sequences for Rattus norvegicus miRNAs (rno-miRNA) from miRbase (release 22.1) and subsequently annotated [29, 30]. Prediction of the novel miRNAs was accomplished using miRDeep2 software (v2.0.0.8) based on the prediction of the secondary structures of the miRNA precursors [31]. DESeq2 (v1.6.3) [32] was used for the analysis of differential expression performed with differentially expressed miRNAs among groups, which were then chosen for further analysis based on p-value <0.05, along with fold change >2. This differentially expressed miRNA target gene prediction relied on the use of computational miRNA sequences along with the associated genomic cDNA sequences, which were determined using Miranda (v3.3a) [31].

### 2.7 Data Interpretation

Target gene candidate *FBF1* functions were searched from genecard.org, along with other review articles and research articles, with the first 50 other target gene candidates of novel-rno-miRNA-1135 chosen from the computational data of authors.

## 3 RESULTS

The results of (Exercise vs. Exercise + PIA) groups provided novel-rno-miRNA-1135 downregulation (-log2), as shown in Table **1**. However, no novel-rno-miRNA-1135 downregulation was observed in other interventional study groups in (N-Exercise + PIA vs. Exercise + PIA), (N-Exercise vs. Exercise), and (N-Exercise vs. N-Exercise + PIA). The novel-rno-miRNA-1135 also targeted 50 other target gene candidates, as shown in Fig. (**2**).

## 4 DISCUSSION

The results showed that *FBF1* was significantly upregulated in the exercise group and downregulated in the novel-rno-miRNA-1135. Regulation of the *FBF1* depends on unknown variable factors, including the gene itself, which has a distinctive expression [33]. This distinguished gene is associated with signaling, age, genetics, individualism, and protein components [33]. The current study focused on a carrier father with *FBF1* mutation related to HYDS1, which possibly conferred the mutation gene to offspring. Many studies have mentioned that the centriole is a direct structure and functions in the appendage distal tip of the ciliogenesis [33, 34]. However, *FBF1* mutation in carrier fathers with possible HYDS 1 inheritance resulted in abnormal formation syndrome due to distal centriole elongation by distal centriole proteins. To build a special protein, centriole elongation forms a specialized construction stage and supports the de novo assembly of TFs, leading to phenotypic malformation of HYDS1 in infants [34]. The results of this study showed that the *FBF1* was upregulated over the novel-rno-miRNA-1135 regulation in the exercise group. The results helped form the assumption in this study that exercise may not be associated with distal centriolar structure and elongation because the *FBF1* might not be changed from exercise.

There are many directions and mechanisms that can affect varied gene expressions, the same as *FBF1* expressions. For the mechanism of *FBF1* up-regulation to the novel rno-miRNA-1135, it could be considered that the gene might be associated with the A-kinase anchoring protein (AKAP9) and that the protein is related to *FBF1* regulation on TFs [33, 35]. However, this mechanism may depend on the environment and physical activity of individualism. The binding of protein partners to the *FBF1* could be associated with the process of exercise and the environment because exercise can change body energy expenditure and mitochondrial biogenesis [33, 35]. Physical activity could downregulate or upregulate both *AKAP9* and *FBF1* following different physical activity levels to change the gene regulations within the nucleus. The AKAP9 protein self-mechanism could also process partner proteins like FBF1 to become expressed or silent. Both compartment protein mechanisms are associated with genetic polymorphisms and/or *AKAP9* mutations [35] that could affect *FBF1* gene expression during exercise. Genomic and epigenetic factors change from physical activity to exercise intensities. For example, *TGFBR3, PDGFD*, *PPM1L, MYC*, *KCNJ2, ST3GAL6, ROPN1L,* and *SLC37A3* were expressed (**1**) before, (**2**) immediately after exercise, and (**3**) after recovery periods of 30 or 60 min [36, 37]. Exercise can also induce the expression of *NDUFA1* and *CASP3,* which are used to predict the respiratory chain component in patients with coronary artery disease [37, 38]. The *FBF1* mechanism may alter its expression during exercise depending on mammal types and ages [34]. One study investigated two- and eight-month-old *Fbf1*^tm-tm^ knockout mice, which showed a decrease in lean body mass compared with wild-type mice [34]. However, it is difficult to identify the correlation between exercise and *FBF1* in terms of how different exercise interventions react to *FBF1* expressions. Further investigations are necessary to address advanced experimentation related to *FBF1* mutations and the exercise process in humans.

The impact of the environment and exercise can change an epigenetic mechanism, such as in the miRNA alternations to make genes silent. The small non-coding miRNAs in mammalian male disease carriers can normally alternate their target gene to become silent, and silencing can confer the gene defects to offspring [39]. Like *FBF1*, the gene could be expressed or silenced from miRNA regulation. However, like other genes, *FBF1* could mutate by DNA alternations, and the gene could be passed on to progeny *via* sperm centriole [40]. The mechanism of miRNA change is associated with external/internal physical body exposure to different environments, durations, and specific exposed tissue types [40]. The results of this study showed that the novel-rno-miRNA-1135 target *FBF1* was downregulated under exercise in the exercise group. Epigenetics, which focuses on miRNA mechanisms, suggests that miRNAs can be upregulated or downregulated by the environment and exercise conditions [40, 41].

The introduction of PIA for rheumatoid in the current investigation might stimulate and influence the *FBF1* to upregulate novel rno-miRNA-1135 regulation. This mechanism suggested the possibility that PIA administration could alter the T-lymphocyte phenotype and its functions, which could consequently affect novel-rno-miRNA-1135 and *FBF1* [42, 43]. In contrast, Nossal (1994) found that T lymphocytes in rats did not respond to any specific antigen [44]. However, these studies could not completely conclude the association of the immune system with *FBF1* and limited the elucidation for unsolved investigations of *FBF1* expression related to exercise.

The findings of this present study hold significant promise as a potential input for the development of future optional medical guidelines for pre-conception screening of fathers. This research may contribute to the identification of specific genetic mutation markers associated with both HYDS1 and, potentially, certain arthritic conditions, including rheumatoid arthritis, all of which share a common underlying gene. Currently, routine screening for such specific genetic biomarkers in fathers, particularly for the purpose of detecting HYDS1 in offspring, remains largely absent from standard medical and healthcare practices. Therefore, the implementation of this novel approach has the potential to become a valuable new protocol within the realm of family planning, ultimately preventing the transmission of inheritable diseases to future generations.

## CONCLUSION

As this examination is a preliminary study, its conclusion can assert that exercise does not have an impact on the *FBF1* becoming down-regulated in the exercise group. The strength of this study is the discovery of the novel-miRNA-1135, down-regulated in an exercise intervention group with *FBF1* gene candidate expressions in the fathers who did not have a rheumatoid condition. However, *FBF1* was not identified for the expression levels because the results herein were based on computerized data and limited by funding constraints. Thus, future research concerning knock-in and knock-out genes in rodents and gene PCR amplification is required.

## Figures and Tables

**Table 1:** Exercise intervention groups for Novel-rno-mi-RNA sequencing H-EX *VS* HE+PIA.

**Rno-miRNA**	**Novel-rno-miRNA-1135**
log2FoldChange	-5.65864
lfcSE	2.678691
p-value	0.034647
Regulation	Down
Sequence	5´ucagaggcaauggaggagcaa 3´

## Data Availability

The data supporting the findings of the article are available in the NCBI Sequence Read Archive (SRA) repository. The data can be accessed *via* the link: https://dataview.ncbi.nlm.nih.gov/object/PRJNA877590?Reviewer=qosgli8l16lvn3qmot5mp11t5g, reference number - BioProject ID: PRJNA877590.

## LIST OF ABBREVIATIONS

AKAP: A-kinase Anchoring Protein
CO_2_: Carbondioxide
EDTA: Ethylnediaminetetraacetic Acid
EX: Exercise without PIA Induction
EX+PIA: Exercise with PIA Induction
*FBF1*: Fas Binding Factor 1
HYDS1: Hydrolethalus Syndrome1
mi-RNA: Micro-Ribonucleic Acid
N-EX: Non-exercise in the Absence of PIA Induction
N-EX+PIA: Non-exercise with PIA Induction
NKT: Natural Killer T-cell
PCR: Real-time Polymerase Chain Reaction
PIA: Pristane-induced Arthritis
PUF: Pumilio
Rno: Rattus Norvegicus
TF: Transition Factor
TZ: Transition Zone
